# Organotins Disrupt the 11β-Hydroxysteroid Dehydrogenase Type 2–Dependent Local Inactivation of Glucocorticoids

**DOI:** 10.1289/ehp.8209

**Published:** 2005-07-14

**Authors:** Atanas G. Atanasov, Lyubomir G. Nashev, Steven Tam, Michael E. Baker, Alex Odermatt

**Affiliations:** 1 Department of Nephrology and Hypertension, Department of Clinical Research, University of Berne, Berne, Switzerland; 2 Department of Medicine, University of California, San Diego, La Jolla, California

**Keywords:** cortisol, dibutyltin, 11β-hydroxysteroid dehydrogenase, glucocorticoid, inhibition, organotin, toxicity, tributyltin, triphenyltin

## Abstract

Organotins, important environmental pollutants widely used in agricultural and industrial applications, accumulate in the food chain and induce imposex in several marine species as well as neurotoxic and immunotoxic effects in higher animals. Reduced birth weight and thymus involution, observed upon exposure to organotins, can also be caused by excessive glucocorticoid levels. We now demonstrate that organotins efficiently inhibit 11β-hydroxysteroid dehydrogenase type 2 (11β-HSD2), converting active 11β-hydroxyglucocorticoids into inactive 11-ketoglucocorticoids, but not 11β-HSD1, which catalyzes the reverse reaction. Di- and tributyltin as well as di- and triphenyltin inhibited recombinant and endogenous 11β-HSD2 in lysates and intact cells with IC_50_ values between 500 nM and 3 μM. Dithiothreitol protected 11β-HSD2 from organotin-dependent inhibition, indicating that organotins act by binding to one or more cysteines. Mutational analysis and 3-D structural modeling revealed several important interactions of cysteines in 11β-HSD2. Cys^90^, Cys^228^, and Cys^264^ were essential for enzymatic stability and catalytic activity, suggesting that disruption of such interactions by organotins leads to inhibition of 11β-HSD2. Enhanced glucocorticoid concentrations due to disruption of 11β-HSD2 function may contribute to the observed organotin-dependent toxicity in some glucocorticoid-sensitive tissues such as thymus and placenta.

Organotins belong to the most widely used organometallic compounds, with an estimated annual production of approximately 50,000 tons. Derivatives of dialkyltin compounds such as dibutyltin (DBT), diphenyltin (DPT), and dioctyltin (DOT) are used in industry as stabilizers in polyvinyl chloride (PVC) and as catalysts in various products, whereas trialkyltins, including tributyltin (TBT) and triphenyltin (TPT) are used in agriculture as fungicides and pesticides and as antifouling agents for large ships (Fent 1996). Organotins are ubiquitous environmental pollutants especially relevant for water ecosystems. Accumulation of these lipophilic compounds has been observed in various species of snails, mussels, and fish (Bhosle et al. 2004; Coelho et al. 2002), causing an increased incidence of sterility or imposex (imposition of male sex characters onto the female) (Evans and Nicholson 2000; Fent 2003).

The main sources of organotin intake for humans are seafood contaminated because of the exposure to antifouling agents (Takahashi et al. 1999), and drinking water contaminated because of the leaching from PVC water pipes (Sadiki and Williams 1999). Additional sources are indoor dust, and liquids stored in plastic containers, including various alcoholic beverages (Liu and Jiang 2002). In higher species, including mammals, organotins tend to accumulate in certain organs, namely liver, kidney, and brain (Fait et al. 1994). Organotins efficiently penetrate through the skin and easily cross the placenta and blood–brain barrier (Adeeko et al. 2003; Cooke et al. 2004; Hasan et al. 1984).

Comparison of the effects of various trialkyltins indicated that the compounds with short alkyl groups such as trimethyltin (TMT) and triethyltin were mainly neurotoxic, whereas organotins with alkyl chains of intermediate length (tripropyltin and TBT) were primarily immunotoxic (Snoeij et al. 1985). The higher trialkyltin homologs trihexyltin and trioctyltin were found to be only slightly toxic; however, further metabolism *in vivo* converted them to their dialkyltin forms, which are also highly immunotoxic (Penninks et al. 1985; Seinen and Willems 1976; Snoeij et al. 1988). A single oral dose of DOT, DBT, or TBT induces a dose-related reduction of the relative thymus weight in rats, and impaired cell-mediated immunity was observed after dietary exposure to TPT for several weeks (Krajnc et al. 1984; Seinen et al. 1977a, 1977b; Snoeij et al. 1988; Vos et al. 1984a, 1984b, 1990). Furthermore, exposure of pregnant rats to organotins causes reduced birth weight (Adeeko et al. 2003; Cooke et al. 2004; Crofton et al. 1989).

Reduced birth weight has also been observed with prolonged intrauterine glucocorticoid exposure (Benediktsson et al. 1993; Lindsay et al. 1996a, 1996b; Stewart et al. 1995). After such an insult, circulating cortisol levels remained elevated throughout adult life, indicating a permanently disturbed regulation of the hypothalamic–pituitary–adrenal axis, which leads to a higher susceptibility for cardiovascular and metabolic disorders including obesity, insulin resistance, and type II diabetes (Drake et al. 2005; Seckl et al. 2000). In the placenta the fetus is protected from the high maternal glucocorticoid concentration through the activity of 11β-hydroxysteroid dehydrogenase type 2 (11β-HSD2), which converts active 11β-hydroxyglucocorticoids (cortisol in human, corticosterone in rodents) into inactive 11-ketoglucocorticoids (cortisone in human, 11-dehydrocorticosterone in rodents) (reviewed in Stewart and Krozowski 1999). Impaired 11β-HSD2 activity, due to mutations or the presence of inhibitors such as glycyrrhetinic acid (GA), strongly correlates with reduced birth weight and metabolic complications in later life of the offspring (Drake et al. 2005; Lindsay et al. 1996b; Odermatt 2004; Seckl et al. 2000).

Moreover, exposure of rats to excessive levels of glucocorticoids causes thymus involution (Schuurman et al. 1992), a phenomenon also evident after exposure to organotins. Treatment of rats with high doses of the 11β-HSD inhibitor GA led to a significant elevation of systemic glucocorticoid levels accompanied by thymocyte apoptosis (Horigome et al. 1999).

Despite the fact that both exposure to excessive levels of organotins and glucocorticoids cause low birth weight and thymus involution in animal models, the impact of organotins on the control of the intracellular availability of glucocorticoids has not been studied. Therefore, we investigated the effect of various organotins on the activities of 11β-HSD1, converting inactive 11-keto-glucocorticoids to active 11β-hydroxyglucocorticoids, and of 11β-HSD2, catalyzing the opposite reaction. We also studied the mechanism of organotin-dependent inhibition of 11β-HSD2.

## Materials and Methods

### Chemicals and reagents.

We purchased [1,2,6,7-^3^H]-cortisol, [2,4,6,7-^3^H]-estrone, and [2,4,6,7-^3^H]-estradiol from Amersham Pharmacia (Piscataway, NJ, USA); [1,2,6,7-^3^H]-cortisone from American Radiolabeled Chemicals (St. Louis, MO, USA); cell culture media and supplements from Invitrogen (Carlsbad, CA, USA); and steroid hormones from Steraloids (Wilton, NH, USA). All other chemicals were obtained from Fluka AG (Buchs, Switzerland) and were of the highest grade available. Organotins were dissolved in dimethyl sulfoxide (DMSO) and stored as 20-mM stock solutions at −70°C. Human 11β-HSD1 and 11β-HSD2 expression constructs in pcDNA3 vector (Invitrogen) were described previously (Odermatt et al. 1999). Plasmids containing cDNA from human 17β-HSD1 or 17β-HSD2, kindly provided by Stefan Andersson, were recloned into pcDNA3 vector by PCR with primers at the 5′ end containing a *Hind*III restriction site (17β-HSD1) or a *BamH*I restriction site (17β-HSD2), a Kozak consensus sequence (Kozak 1989) and the initiation codon, and primers at the 3′ end containing a stop codon followed by an *Xba*I restriction site. All constructs were verified by sequencing.

### Cell culture.

HEK-293 (human embryonic kidney) cells stably transfected with FLAG (Asp–Tyr–Lys–Asp–Asp–Asp–Asp–Lys)-tagged human 11β-HSD2 (Schweizer et al. 2003) were grown at 37°C under 5% carbon dioxide to 60–70% confluence in Dulbecco’s modified Eagle medium (DMEM) supplemented with 10% fetal calf serum, 4.5 g/L glucose, 50 U/mL penicillin/streptomycin, and 2 mM glutamine. SW-620 (human colorectal adenocarcinoma) cells and JEG-3 (human choriocarcinoma) cells were cultured according to the recommendations of the supplier (American Type Culture Collection, Manassas, VA, USA). The recently described RCCD-2 aldosterone-sensitive rat cortical collecting duct cells (Djelidi et al. 2001) were cultured in DMEM/Ham’s F-12 (1:1), 14 mM NaHCO_3_, 2 mM glutamine, 10 U/mL penicillin/streptomycin, and 20 mM HEPES, pH 7.4.

### Activity assays in cell lysates.

To measure 11β-HSD2 activity, stably transfected HEK-293 cells (Schweizer et al. 2003) were grown in 10-cm culture dishes to 90% confluence. Cells were rinsed 3 times with phosphate-buffered saline (PBS) and resuspended in 2 mL ice-cold buffer TS2 (100 mM NaCl, 1 mM EGTA, 1 mM EDTA, 1 mM MgCl_2_, 250 mM sucrose, 20 mM Tris-HCl, pH 7.4). Aliquots of the cell suspension were frozen at −20°C, retaining full enzymatic activity for at least 3 months. For determinationof 11β-HSD2 activity, aliquots were thawed, sonicated, and diluted 1:12 in buffer TS2 (4°C). We carried out reactions in a final volume of 20 μL containing 10 nCi [1,2,6,7-^3^H]-cortisol, 400-μM NAD^+^, and different concentrations of unlabeled cortisol. Final cortisol concentrations were 40 nM for measurements of inhibitors and ranged between 10 nM and 200 nM for determination of apparent *K*_m_ values. Incubations were for 10 min at 37°C.

For determination of 11β-HSD1, 17β-HSD1, and 17β-HSD2 activity, HEK-293 cells transfected by the calcium-phosphate precipitation method were harvested 48 hr later, washed with PBS, and centrifuged for 3 min at 150 × *g*. Supernatants were removed, cell pellets quick-frozen in a dry ice ethanol bath, and stored at −70°C. For assaying 11β-HSD1, we dissolved pellets in TS2 buffer; for 17β-HSD1 or 17β-HSD2, we used a buffer containing 50 mM potassium phosphate, 20% glycerol, and 1 mM EDTA. We measured 11β-HSD1 oxoreductase activity as described recently (Atanasov et al. 2004), using radiolabeled cortisone as substrate. 17β-HSD1 and 17β-HSD2 activities were measured in the presence of radiolabeled estrone or estradiol and unlabeled steroid at final concentrations of 200 nM and 500 μM NADPH or NAD^+^, respectively.

### Determination of 11β-HSD2 activity in intact cells and MTT cytotoxicity assay.

HEK-293 cells stably transfected with 11β-HSD2 (25,000 cells per well) were seeded 24 hr prior to the assay in poly-d-lysine coated 96-well Biocoat plates (Becton Dickinson, Basel, Switzerland). The medium was carefully removed, followed by the addition of 30 μL fresh medium, 10 μL medium containing various concentrations of organotins, and 10 μL radiolabeled cortisol. The reaction volume was 50 μL, with a final cortisol concentration of 40 nM. The cells were incubated for 2 hr at 37°C under 5% CO_2_. We stopped reactions by adding an excess of unlabeled cortisone and cortisol in methanol, and separated steroids using thin-layer chromatography, followed by scintillation counting. 11β-HSD2 activity in JEG-3, SW-620, and RCCD-2 cells was measured similarly by adjusting the cell density and reaction time to obtain a maximal conversion of cortisol between 15 and 25%.

To ensure that the observed inhibition of 11β-HSD2 activity was not due to cell death, we assessed cytotoxicity of organotin compounds parallel to the activity assay under identical conditions. The corresponding organotin compound was added to the cells, followed by incubation for 2 hr at 37°C under 5% CO_2_. Cells were washed with PBS and incubated in fresh medium containing 0.5 mg/mL 3-(4,5-dimethylthiazol-2-yl)-2,5-diphenyltetrazolium bromide (MTT). After conversion of MTT, we removed the medium and added 200 μL DMSO to the insoluble fraction. Conversion of MTT was kept below OD 0.9 ^(^*^A^*^570–^*^A^*^690)^.

### Site-directed mutagenesis and analysis of mutant 11β-HSD2 enzymes.

Mutations were introduced into the C-terminally FLAG-tagged 11β-HSD2 cDNA in Bluescript vector by site-directed mutagenesis according to the Quick Change mutagenesis kit (Stratagene, Amsterdam, the Netherlands) (Odermatt et al. 1999). All constructs were verified by sequencing and recloned into pcDNA3 expression vector. Wild-type and mutant enzymes were expressed in HEK-293 cells, lysates were prepared, and proteins were separated by sodium dodecyl sulfate (SDS) gel electrophoresis. Proteins were transferred to nitrocellulose, and expression levels of 11β-HSD2 constructs were detected using mouse monoclonal antibody M2 raised against the FLAG epitope and visualized with a horseradish peroxidase conjugated anti-mouse antibody and enhanced chemiluminescence Western kit (Pierce, Rockford, IL, USA). After detection of 11β-HSD2 constructs, nitrocellulose membranes were stripped and incubated with rabbit polyclonal anti-actin IgG (Santa Cruz Biotechnology Inc., Santa Cruz, CA, USA) and horseradish peroxidase–conjugated goat anti-rabbit IgG to adjust for the amount of proteins loaded on the gel. The expression of mutant relative to wild-type enzyme was adjusted for calculation of kinetic parameters.

### Three-dimensional modeling.

The 3-D model of human 11β-HSD2 from Arnold et al. (2003) was used with NAD^+^ extracted from protein data bank file 1AHI (Tanaka et al. 1996). Human 11β-HSD2 was then minimized for 100,000 iterations with Discover 3 (Accelrys Inc., San Diego, CA, USA) using the extensible and systematic force field (ESFF), with a distant dependent dielectric constant of 2, to model water in the protein.

### Statistical analysis.

Enzyme kinetics were analyzed by nonlinear regression using Data Analysis Toolbox (MDL Information Systems Inc., Nashville, TN, USA) assuming first-order rate kinetics. Data represent mean ± SD of at least four independent experiments.

## Results

### Inhibition of 11β-HSD2 but not 11β-HSD1 by organotins.

To investigate whether organotins disrupt the control of the ratio of active to inactive glucocorticoids by inhibition of 11β-HSD enzymes, we incubated lysates of HEK-293 cells expressing recombinant 11β-HSD constructs with various concentrations of TBT and TPT and determined enzymatic activities. TBT and TPT did not inhibit the 11β-HSD1–dependent conversion of cortisone to cortisol at concentrations up to 200 μM (Table 1). In contrast the 11β-HSD2–dependent conversion of cortisol to cortisone was efficiently inhibited by both compounds with IC_50_ (median inhibitory concentration) values in the low micromolar range, indicating that organotins selectively abolish the 11β-HSD2–dependent inactivation of glucocorticoids.

Despite that 11β-HSD1 and 11β-HSD2 interconvert the same substrate, they are phylogenetically relatively distant enzymes, sharing only 18% identical amino acid sequence (Baker 2004). 11β-HSD2 is more closely related to 17β-HSD2, with an amino acid sequence about 45% identical. Thus, we also determined the conversion of estrone to the more potent estrogen estradiol by 17β-HSD1 and the reverse reaction by 17β-HSD2 in the presence of various concentrations of organotins. As shown in Table 1, neither TBT nor TPT inhibited 17β-HSD1. 17β-HSD2 activity was inhibited by TPT at a 6-fold higher IC_50_ value compared with that of 11β -HSD2, indicating that organotins preferentially inhibit 11β-HSD2.

Because trialkyltins are progressively dealkylated by microorganisms in the environment and in mammalian organs including brain, liver, and kidneys (Gadd 2000), and because the dialkyltins DBT and DPT are major metabolites with significant toxicity (Penninks et al. 1985; Seinen and Willems 1976; Snoeij et al. 1988), we included these two compounds in the study. Analysis of all four organotin compounds revealed comparable inhibitory properties both in lysates and intact cells expressing 11β-HSD2 with IC_50_ values in the low micromolar range (Table 1). In intact cells the trialkyltins were approximately 3-fold more potent than the dialkyltins, and the phenyltins were slightly more potent than the butyltins, well correlating with the hydrophobicity of these compounds. It is important to note that the inhibitory potency of organotins is comparable with that of the well-known 11β-HSD inhibitor GA in intact cells.

### Dithiothreitol but not glutathione protects 11β-HSD2 from inhibition by organotins.

**Figure 1:**
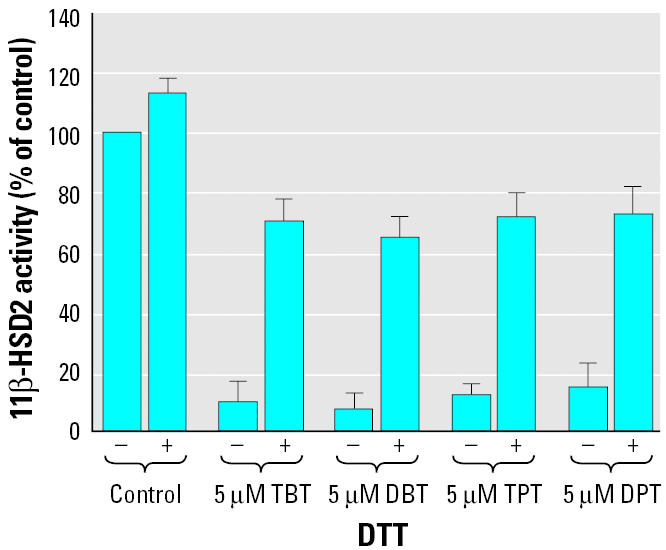
DTT prevents organotin-dependent inhibition of 11β-HSD2. The oxidation of cortisol by 11β-HSD2 was determined using cell lysates, as described in “Materials and Methods.” Addition of DTT at a final concentration of 2 mM restored 70–80% of the activity measured in absence of organotins.

To investigate the molecular mechanism of organotin-induced inhibition of 11β-HSD2, we measured the effect of organotins in the presence or absence of the reducing agent dithiothreitol (DTT) (Figure 1). DTT alone did not significantly alter enzymatic activity. A concentration of 2 mM DTT, added simultaneously with 5 μM of the corresponding organotin compound, restored 70–80% of 11β-HSD2 activity. Upon preincubation of 11β-HSD2 with organotins for 15 min, 50–60% of the enzymatic activity could be restored (not shown), indicating that most but not all of the inhibitory effect was reversible. In contrast to the dithiol DTT, the monothiol glutathione was not able to prevent organotin-dependent inhibition of 11β-HSD2 (not shown).

We recently demonstrated that dithiocarbamates, another environmentally relevant class of compounds, inhibit 11β-HSD2. Cofactor NAD^+^ partially protected the enzyme, suggesting that covalent modification of the thiol group at Cys^90^ in the cofactor-binding region may be responsible for dithiocarbamate-induced inactivation of 11β-HSD2 (Atanasov et al. 2003). In contrast we found no protective effect of NAD^+^ on organotin-induced enzyme inhibition (not shown), indicating an inhibitory mechanism distinct from that of dithiocarbamates.

### Functional analysis of the cysteine residues of 11β-HSD2.

**Figure 2:**
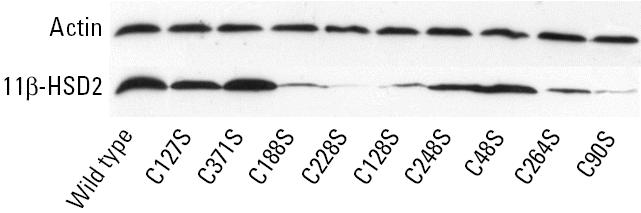
Expression of wild-type 11β-HSD2 and cysteine to serine mutants. C-terminally FLAG-epitope tagged wild-type and mutant 11β-HSD2 enzymes were expressed in HEK-293 cells, and protein expression was analyzed by Western blotting as described in “Materials and Methods.” After detection of the FLAG-tagged 11β-HSD2 enzymes, nitrocellulose membranes were stripped, and actin expression was detected as a control for the amount of protein loaded on the SDS gel. A representative blot from three comparable experiments is shown.

We have previously shown that substitution of Cys^90^ by serine leads to abolished protein expression and enzymatic activity (Atanasov et al. 2003). Because NAD^+^ did not protect 11β-HSD2 from organotin-induced inhibition, we analyzed the functional relevance of the remaining eight cysteine residues by mutating them to serines. All nine cysteine-to-serine mutants contained a FLAG epitope at the C-terminus, allowing the quantification of the relative protein expression (Figure 2). Five of the mutant enzymes showed significantly reduced expression. No band could be detected for mutant Cys^228^Ser and only a very weak band for Cys^90^Ser was detected. As expected, no activity could be measured using lysates from cells transfected with cDNA for either of these two mutant enzymes. Significantly reduced expression was also observed for mutants Cys^128^Ser and Cys^188^Ser. When the catalytic activity of these mutant enzymes was adjusted for their reduced expression, an apparent *V*_max_ comparable to that of wild-type 11β-HSD2 was obtained (Table 2), indicating reduced enzyme stability but intact catalytic activity. For mutant Cys^264^Ser, which also showed reduced expression, a slightly higher *V*_max_ was obtained. Comparison of the kinetic parameters revealed altered kinetic parameters for mutant Cys^264^Ser, with a 3-fold higher apparent *K*_m_. Mutation of Cys^264^ to serine also resulted in a 2-fold increase in the IC_50_ for TBT, suggesting a role for Cys^264^ in the binding of cortisol and in the interaction with organotins with 11β-HSD2.

### Analysis of the mode of organotin-dependent inhibition of 11β-HSD2.

**Figure 3:**
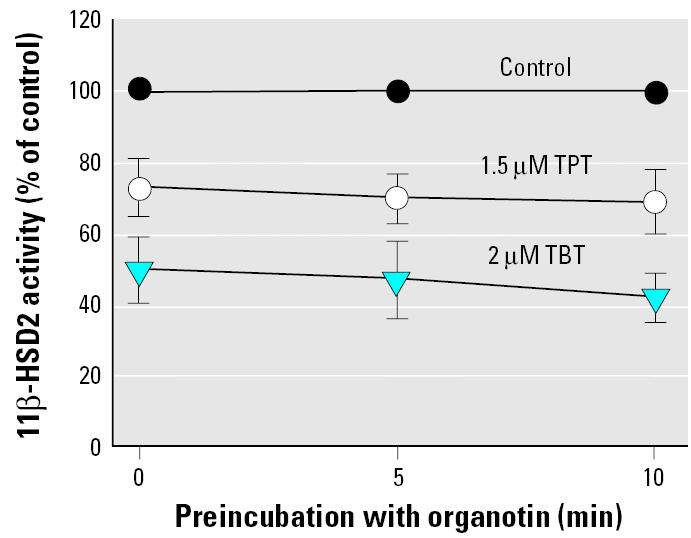
Effect of preincubation on 11β-HSD2 activity. The oxidation of cortisol to cortisone was determined after preincubation for 5 or 10 min with vehicle (control), 1.5 μM TPT, or 2 μM TBT in lysates of HEK-293 cells expressing 11β-HSD2. Data are mean ± SD from at least three independent experiments.

We next investigated the effect of preincubation of 11β-HSD2 with either 1.5 μM TPT or 2 μM TBT for 5 or 10 min. Although there was a slight tendency toward increased inhibitory effect upon preincubation with TBT, the changes did not reach significance (Figure 3), in line with reversible inhibition. To further assess the mode of inhibition, we determined the change of apparent *K*_m_ and *V*_max_ in the absence or presence of TBT. The apparent *K*_m_ increased 2- and 3-fold upon incubation with 2 μM and 3 μM TBT (62 ± 17 nM for the control compared with 137 ± 24 nM and 197 ± 33 nM for treated samples, respectively), whereas *V*_max_ decreased slightly (2.14 ± 0.32 nmol × h^−1^ × mg^−1^ for the control compared with 1.66 ± 0.40 nmol × h^−1^ × mg^−1^ and 1.48 ± 0.48 nmol × h^−1^ × mg^−1^ for treated samples, respectively). These findings suggest a mixed-competitive mode of inhibition, with most of the inhibitory effect being reversible.

**Figure 4:**
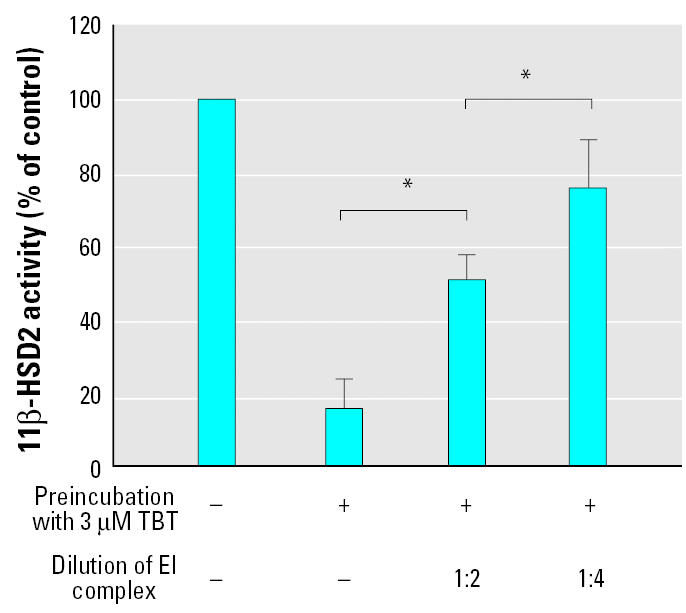
Effects of dilution of the enzyme-inhibitor (EI) complex on TBT-dependent inhibition of 11β-HSD2. Lysates from HEK-293 cells expressing 11β-HSD2 were split into two equal aliquots. TBT was added to the first aliquot, and the same volume of vehicle, serving as a control, was added to the second. Both aliquots were incubated for 5 min at 37°C, followed by determination of the activity of the control and EI mixture either undiluted or after a 2- or 4-fold dilution. Data are mean ± SD from at least three independent experiments measured in triplicate. *p < 0.05.

To further test this assumption, we measured the effect of diluting the enzyme–inhibitor complex (EI) (Figure 4). In case of a competitive mode of inhibition, dilution of the EI complex would lead to a decreased inhibitor concentration and reduced competition with the substrate, hence reduced enzyme inhibition. If covalent enzyme modification takes place, the dilution of the EI complex would not change the proportion of modified to unmodified molecules, meaning that after the dilution of EI there would be no change of the relative inhibition compared with the control. In case of TBT-induced inhibition of 11β-HSD2, a 2-fold and 4-fold dilution of the EI complex led to a proportional decrease of the relative inhibitory effect, indicating a transient interaction of the organotin compound with 11β-HSD2.

### Inhibition of 11β-HSD2 in endogenous cell lines.

**Figure 5:**
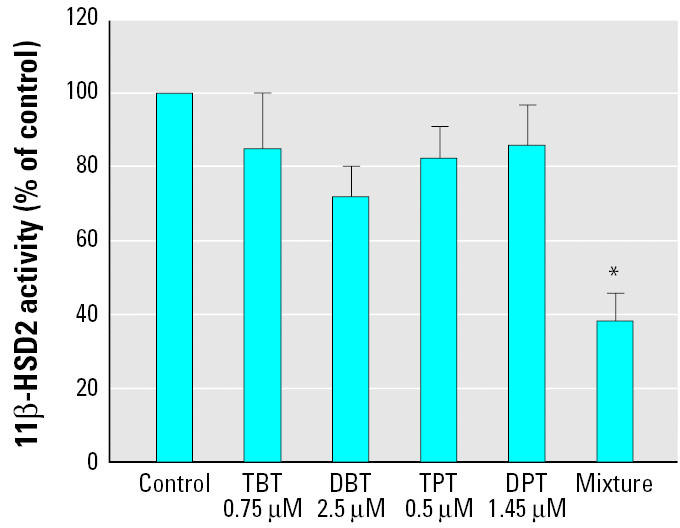
Additive inhibitory effect of a mixture of organotins on 11β-HSD2 activity. Conversion of cortisol to cortisone by 11β-HSD2 stably expressed in intact HEK-293 cells was measured in a volume of 50 μL cell culture medium containing 40 nM cortisol and the corresponding amount of the organotin, as indicated (see “Materials and Methods”). Data were normalized to the control and are mean ± SD from at least three independent experiments measured in triplicate. *Statistical significance of *p* < 0.01 compared with all other values.

Because organisms are exposed to various sources of organotins and these chemicals undergo dealkylation *in vivo*, cells in tissues are generally exposed to a mixture of organotins. Therefore, we compared the activity of 11β-HSD2 in intact cells either upon incubation with DBT, TBT, DPT, or TPT alone, at concentrations 50% below their IC_50_ value, or after incubation with a mixture of the four chemicals (Figure 5). Whereas each compound alone reduced 11β-HSD2 activity only slightly, the mixture showed additive inhibitory effects and significantly inhibited enzymatic activity, indicating that the distinct organotins act by the same mechanism. We observed this phenomenon in transfected HEK-293 cells as well as in endogenous cell lines.

**Figure 6:**
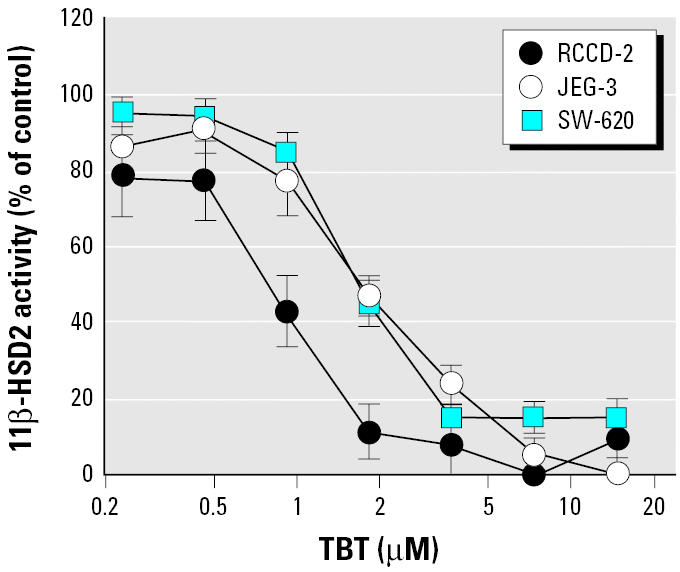
Dose–response curves for TBT-induced inhibition of 11β-HSD2 in intact cells expressing endogenous 11β-HSD2. RCCD-2, rat renal cortical collecting duct cell line; JEG-3, human choriocarcinoma cell line; SW-620, human colon adenocarcinoma cell line. Details on culture conditions and activity assay in intact cells are given in “Materials and Methods.”

We next determined the potential of TBT to inhibit 11β-HSD2 activity in cell lines derived from tissues with endogenous expression of this enzyme, for example, placenta, renal cortical collecting duct, and colon (Figure 6). In placenta-derived JEG-3 cells and in colon-derived SW-620 cells, the inhibition of 11β-HSD2 by TBT was highly similar to that observed in HEK-293 cells. We observed approximately 2-fold stronger inhibition in renal cortical-collecting duct–derived RCCD-2 cells, with an IC_50_ of 0.83 ± 0.23 μM for TBT.

## Discussion

Relatively little is known about the molecular targets of organotins despite their wide range of toxic effects and that they can be readily detected in the blood of humans. In this article we describe the organotin-dependent inhibition of 11β-HSD2. Our data suggest that organotins inhibit 11β-HSD2 by a mostly reversible, mixed-competitive mode of inhibition. Comparison of the kinetic parameters obtained from measurements with lysates and intact cells expressing 11β-HSD2 indicates that the trialkyltins enter the cell more easily, which explains their more potent effects in intact cells. However, dialkyltins display equal or even enhanced inhibitory potency in lysates. Organotin-induced inhibition of 11β-HSD2 was prevented by the dithiol DTT but not by the endogenous monothiol glutathione, which sugggests that two cysteine residues in close proximity might be involved in the mechanism of inhibition. This is in contrast to the inhibition of 11β-HSD2 by dithiocarbamates, which irreversibly inhibit the enzyme, probably through covalent modification of a cysteine residue by attachment of a carbamoyl group (Atanasov et al. 2003).

**Figure 7:**
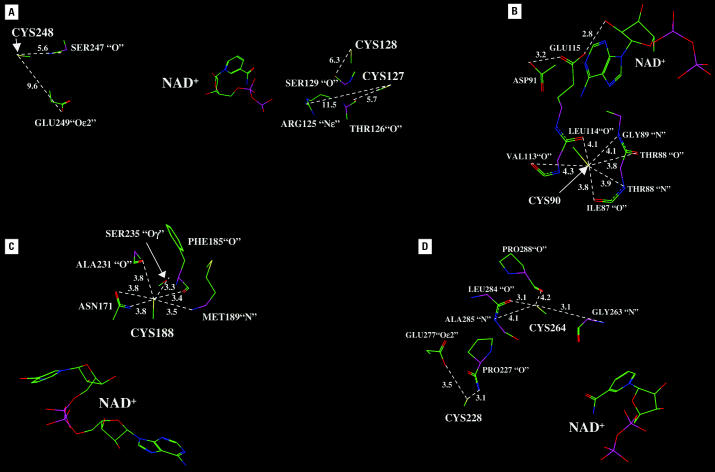
Predicted interactions of cysteine residues in the conserved core domain of 11β-HSD2. ( A) Cys^127^, Cys^128^, and Cys^248^ are oriented into the solvent, away from the catalytic pocket, and have few stabilizing interactions with other residues. ( B) Cys^90^ interacts with amino acids that stabilize Glu^115^ and Asp^91^, which have a critical role by forming hydrogen bonds to the ribose hydroxyl on NAD^+^ that are important to stabilize binding of the cofactor and maintain its orientation to the substrate. ( C) Cys^188^ is not directly involved in interactions with NAD^+^ but stabilizes several amino acids that interact with the pyrophosphate segment of the cofactor. ( D) The thiol on Cys^228^ stabilizes the position of Pro^227^ and Glu^277^, which are important for positioning of the catalytic tyrosine and the nicotinamide ring and for binding of the steroid substrate. The thiol group of Cys^264^ has important interactions with Leu^284^, Ala^285^, and Pro^288^ in the helix in the C-terminal region of 11β-HSD2, which is important for substrate binding. Predicted interatomic distances in angstroms are depicted by number. Blue: nitrogen; green: carbon; purple: phosphorus; red: oxygen; and yellow: sulfur.

The inhibition of 11β-HSD2 by dithiocarbamates (Atanasov et al. 2003) and organ-otins seems to involve distinct cysteine residues, as addition of high concentrations of cofactor NAD^+^ partially protected from dithiocarbamates but not from organotins. Site-directed mutagenesis and functional analysis revealed an essential role of Cys^128^, Cys^188^, Cys^228^, and Cys^264^ for enzyme stability and/or catalytic activity in addition to the previously described Cys^90^. To begin to understand the basis for the different activities of these mutant enzymes, we applied a 3-D structural model of 11β-HSD2 (Arnold et al. 2003; Atanasov et al. 2003) to investigate the structure surrounding each of these cysteines (Figure 7). Cys^48^ and Cys^371^ are located in hydrophobic segments thought to associate with cellular membranes in the endoplasmic reticulum. These residues are outside the 260 residue core segment comprising the catalytically active domain in homologs of 11β-HSD2 and were not analyzed further. The 3-D model shows that Cys^127^, Cys^128^, and Cys^248^ do not interact with sites on 11β-HSD2 critical for binding of either NAD^+^, substrate, or the catalytically active Tyr^232^. Figure 7A shows that these cysteines have few stabilizing interactions with other amino acids and are oriented to the solvent, away from the catalytic pocket.

As described previously (Atanasov et al. 2003), the thiol group of Cys^90^ has several interactions with amino acids that stabilize Glu^115^ and Asp^91^ (Figure 7B). Glu^115^ has critical hydrogen bonds with the ribose hydroxyl on NAD^+^ that are important in stabilizing binding of the cofactor and in maintaining its orientation to the substrate. Thus, the loss of the stabilizing effects of Cys^90^ on the orientation of Glu^115^ leads to an inactive 11β-HSD2.

Similarly, the thiol group on Cys^188^ stabilizes several amino acids that interact with the pyrophosphate segment of NAD^+^ (Figure 7C). However, like Cys^90^, Cys^188^ is not directly involved in interactions with the cofactor, and it appears that if the thiol group is replaced with a serine hydroxyl group, there remains sufficient stabilization of the structure near the pyrophosphate group in mutant Cys^188^Ser to retain some catalytic activity.

Cys^228^ is in the loop that precedes the catalytically active Tyr^232^ (Figure 7D). Evidence from solved 3-D structures of 11β-HSD2 homologs, such as 17β-HSD1, clearly shows that this loop stabilizes the position of the nicotinamide ring and the catalytic Tyr^232^ with the steroid substrate to promote catalysis (Breton et al. 1996; Ghosh et al. 1994; Tanaka et al. 1996; Yamashita et al. 1999). The thiol on Cys^228^ stabilizes the backbone oxygen in Pro^227^, which is an important structural amino acid that is also in the loop that positions the catalytic tyrosine, the nicotinamide ring, and the steroid. Cys^228^ also stabilizes Glu^277^, which belongs to a helix in the substrate binding site. Thus, the thiol group on Cys^228^ interacts with amino acids on 11β-HSD2 that are important in substrate binding, which explains the loss of activity of mutant Cys^228^Ser.

Figure 7D also shows that the thiol group of Cys^264^ has important interactions with Leu^284^, Ala^285^, and Pro^288^, which are part of the helix in the C-terminal region of 11β-HSD2 that likely is important in the substrate binding site, based on analyses of 17β-HSD2 and other homologs (Tanaka et al. 1996) of 11β-HSD2. Our experimental data indicate that the serine hydroxyl group can partially take over the function of the thiol on Cys^264^. Together, these findings suggest that Cys^228^ and Cys^264^ may be involved in the interactions with organotins, and interference with the function of their thiol groups may be responsible for inactivation of 11β-HSD2 by organotins.

Although the chemical nature of organotin–protein interactions is not completely understood at present, it is believed that most of the properties of organotins are a result of the nature of C–Sn bonds that can be attacked by both nucleophilic and electrophilic reagents (Hoch 2001). Buck et al. recently described the interaction of organotins with the membrane protein stannin (Buck et al. 2003, 2004). A nonapeptide derived from stannin containing the sequence Cys–Trp–Cys was able to dealkylate TMT, followed by binding of the resulting DMT. Buck et al. (2003, 2004) further showed that DTT bound TMT without inducing dealkylation. Stannin efficiently dealkylated organotins with short alkyl side chains with, weak binding observed for TBT and TOT.

11β-HSD2 does not possess a Cys–Xaa–Cys motif, and Cys^127^ and Cys^128^ are unlikely to be the target residues for inhibition by organotins, as replacement of either of these residues did not affect the organotin-dependent inhibition of the mutant enzymes. Replacing Cys^228^ by serine completely abolished enzyme activity, indicating an important functional role of this residue. Mutating Cys^264^ to serine led to a well-expressed enzyme with only slightly reduced catalytic efficiency. This mutant was less sensitive to organotin-dependent inhibition.

The potency of organotins in inhibiting 11β-HSD2 is equal to or greater than that reported for other enzymes involved in steroid hormone metabolism, including cytochrome P450 aromatase (Lo et al. 2003), 5α-reductases (Doering et al. 2002), and rat testis microsomal 3β-HSD (McVey and Cooke 2003). As mentioned earlier, organotins represent ubiquitous contaminants of the water ecosystem. Although most often present at lower nanomolar concentrations, organotins accumulate in aquatic organisms, with up to 70,000 times higher organotin concentrations in plankton and other organisms than in sea water (Takahashi et al. 1999). As little as 200 g of contaminated shell-fish could be sufficient to reach the toxic daily intake of TBT (0.25 μg/kg body weight) (McVey and Cooke 2003). Another major source of organotins for humans is the drinking water in locations where PVC water pipes are used. A study of organotin levels in Canadian drinking water distributed through PVC pipes in 1996 revealed total concentrations of different organotins < 50 ng Sn/L, although in some occasions values > 250 ng Sn/L were detected (Sadiki and Williams 1999). Although the IC_50_ values of organotins for 11β-HSD2 in the present study were in the high nanomolar and low micromolar range, the accumulation of organotins in specific organs and tissues including brain, liver, and kidneys may lead to high local concentrations, as has been found in aquatic organisms. In addition Chicano et al. (2001) provided evidence that organotins are located in the upper part of the phospholipid palisade near the lipid–water interface and affect the degree of hydration of the phospholipid carbonyl moiety. Thus, intracellular organotin concentrations might be highest at the surface of membranes, and 11β-HSD2 might be exposed to concentrations much higher than those in the solution.

The increased glucocorticoid-mediated effects due to the inhibition of 11β-HSD2 are expected to disturb several essential physiologic processes. The effect of TBT and its major metabolite DBT to suppress T-cell–dependent immune functions by causing thymus atrophy has been extensively studied (Krajnc et al. 1984; Seinen et al. 1977a, 1977b; Snoeij et al. 1988; Tryphonas et al. 2004; Vos et al. 1984a, 1990). A single dose of TBT, DBT, or DOT induced dose-dependent reductions in the weight of the thymus, spleen, and lymph node (Seinen et al. 1977b). The effect of TBT was less pronounced and slightly delayed compared with DBT, indicating that *in vivo* TBT is metabolized to the more toxic DBT (Snoeij et al. 1988). The thymotoxic effects of organotins are completely reversible (Seinen et al. 1977b). A selective inhibition of the proliferation of immature CD4^−^/CD8^+^ thymocytes by organotins seems to be responsible for the observed depletion of CD4^+^/CD8^+^ thymocytes, which show a rapid turnover.

11β-HSD enzymes play a pivotal role in regulating proliferation and differentiation in various tissues. 11β-HSD1 generates active glucocorticoids and promotes differentiation, and 11β-HSD2 inactivates glucocorticoids, thereby promoting proliferation. 11β-HSD1 and 11β-HSD2 were both expressed in whole mouse thymus (Moore et al. 2000; Speirs et al. 2004), although the exact subtype-specific expression pattern of 11β-HSD enzymes remains to be determined. In the acute stress response, the high level of glucocorticoids induces thymus involution (Schuurman et al. 1992). Organotin-dependent inhibition of 11β-HSD2 may cause antiproliferative effects on immature thymocytes by increasing locally the ratio of active to inactive glucocorticoids or, alternatively, by increasing systemic glucocorticoid levels. Both organotin-induced inhibition of 11β-HSD2 and thymotoxicity are reversible.

Experiments in mice showed maximal thymocyte apoptosis 8 hr after glucocorticoid administration, followed by full recovery after 18 hr (Ishii et al. 1997). There was a significant depletion of CD4^+^/CD8^+^ thymocytes. A delayed, dose-dependent apoptosis of thymocytes, reaching maximal effect after 24 hr, was observed when mice were treated with a single dose of the 11β-HSD inhibitor GA (Horigome et al. 1999). Thymocyte apoptosis was induced dose-dependently by corticosterone *in vitro*. GA alone did not induce apoptosis *in vitro*, suggesting that elevated corticosterone levels due to inhibition of 11β-HSD2 may cause the apoptosis. Similarly, organotin-dependent inhibition of 11β-HSD2 and subsequent locally enhanced glucocorticoid levels may contribute to the immunotoxicity of these compounds. However, thymus atrophy was also observed in DBT- and TBT-treated adrenalectomized rats (Seinen and Willems 1976; Snoeij et al. 1985), and no extensive cell destruction was observed in DBT- and TBT-treated rats compared with rats treated with very high glucocorticoid concentrations (La Pushin and de Harven 1971). These findings suggest that the immunotoxic effects of organotins are caused by a glucocorticoid-dependent and a glucocorticoid-independent mechanism.

The glucocorticoid-dependent effects caused by organotins may be most critical during pregnancy, where fetal development is highly sensitive to glucocorticoids (Seckl et al. 2000). The lower birth weight and decreased weight gain in the offspring after exposure of organotins during pregnancy is a phenotype also observed as a result of exposure to excessive levels of glucocorticoids. Enhanced glucocorticoid action due to inhibition of 11β-HSD2, which in the placenta protects the fetus from high maternal levels, or due to treatment with synthetic glucocorticoids that cannot be inactivated by 11β-HSD2, such as dexamethasone (Rebuffat et al. 2004), have been associated with reduced birth weight and irreversible changes in the cardiovascular system with complications in later life (Seckl et al. 2000). Thus, inhibition of 11β-HSD2 may be one reason that offspring from pregnant rats treated with organotins show significantly reduced birth weight (Adeeko et al. 2003; Cooke et al. 2004; Crofton et al. 1989).

## Conclusions

This work demonstrates the disruption of the 11β-HSD2–dependent inactivation of glucocorticoids by organotins. Various organotin compounds inhibit 11β-HSD2, mainly by a reversible mode of inhibition, and show additive effects. Endogenous glutathione cannot prevent the organotin-induced inhibition of 11β-HSD2, which explains the comparable inhibitory kinetics obtained in experiments with cell lysates and in intact cells. The results suggest that enhanced glucocorticoid concentrations due to disruption of 11β-HSD2 function may contribute to the observed organotin-dependent toxicity in glucocorticoid sensitive tissues such as thymus and placenta. Clearly, additional experiments *in vivo* must be performed to elucidate the relevance of organotin-dependent interference with glucocorticoid action and its pathophysiologic consequences.

## Figures and Tables

**Table 1:** Selective inhibition [IC_50_ (μM)] of 11β -HSD2 by organotins.^a^

	Cell lysate				
Organotin	17β -HSD1	17β -HSD2	11β -HSD1	11β -HSD2	Intact cells 11β -HSD2
TBT	> 200	> 200	> 200	1.90 ± 0.66	1.52 ± 0.43
DBT	ND	ND	ND	1.95 ± 0.27	5.03 ± 0.70
TPT	> 200	19 ± 3	> 200	3.19 ± 0.73	0.99 ± 0.24
DPT	ND	ND	ND	1.42 ± 0.17	2.89 ± 0.59
GA	ND	ND	ND	0.40 ± 0.08	1.01 ± 0.29

ND, not determined.

a Data are mean ± SD from at least four independent experiments measured in duplicate.

**Table 2:** Analysis of the kinetic parameters of wild-type 11β-HSD2 and cysteine to serine mutants.^a^

	*K*_m_ (nM)	*V*_max_ (nmol × h^−1^ × mg^−1^)	TBT [IC_50_ ± SD (μM)]
Wild-type	62 ± 17	2.14 ± 0.32	1.90 ± 0.46
Cys^48^Ser	71 ± 23	1.93 ± 0.17	2.11 ± 0.33
Cys^90^Ser	ND	ND	ND
Cys^127^Ser	67 ± 19	2.48 ± 0.36	2.23 ± 0.33
Cys^128^Ser	97 ± 21	3.02 ± 0.28	1.87 ± 0.53
Cys^188^Ser	49 ± 16	2.78 ± 0.37	1.95 ± 0.38
Cys^228^Ser	ND	ND	ND
Cys^248^Ser	51 ± 13	2.49 ± 0.43	2.07 ± 0.47
Cys^264^Ser	173 ± 32	3.29 ± 0.35	4.20 ± 0.44
Cys^371^Ser	74 ± 20	2.08 ± 0.28	2.17 ± 0.50

ND, not determined.

a Data are mean ± SD from at least three independent experiments measured in triplicate.
